# Mitigating PM2.5-induced skin injury and aging: botanical strategies targeting redox and inflammatory pathways

**DOI:** 10.1080/13510002.2026.2629079

**Published:** 2026-02-19

**Authors:** Phetthinee Maunjumpon, Onusa Thamsermsang, Uraiwan Panich

**Affiliations:** a Department of Pharmacology, Faculty of Medicine Siriraj Hospital, Mahidol University, Bangkok, Thailand; b Department of Pharmaceutical Sciences, Faculty of Pharmacy, Chiang Mai University, Chiang Mai, Thailand

**Keywords:** Particulate matter ≤ 2.5 μm (PM 2.5), botanicals, phytochemicals, skin injury, redox, inflammation

## Abstract

Exposure to fine particulate matter smaller than 2.5 μm in diameter (PM2.5) has emerged as a critical environmental factor contributing to skin injury. As the skin is the body’s primary barrier against the external environment, it is directly susceptible to PM2.5, which induces oxidative stress, inflammation, premature aging, and disruption of skin barrier function. Increasing evidence demonstrates that PM2.5 damages both epidermal keratinocytes and dermal fibroblasts, leading to cellular dysfunction through alterations in major signaling pathways, including the aryl hydrocarbon receptor (AhR), nuclear factor kappa B (NF-κB), activator protein 1 (AP-1), mitogen-activated protein kinase (MAPK), and nuclear factor erythroid 2–related factor 2 (Nrf2). These molecular perturbations accelerate skin aging and impair protective functions, highlighting the need for effective intervention strategies. Botanicals and their bioactive phytochemicals have attracted growing interest for their antioxidant and anti-inflammatory properties, which may counteract PM2.5-induced damage. By targeting redox imbalance and inflammatory signaling, natural compounds represent a promising approach for protecting skin health. This review highlights the role of PM2.5 in skin injury and critically examines botanical strategies that may mitigate PM2.5-induced skin damage and premature aging.

## 1. Introduction

The World Health Organization (WHO) identifies particulate matter (PM) as a major air pollutant, including fine particles such as PM2.5 and PM10, which are classified based on their aerodynamic diameters of less than 2.5 µm and 10 µm, respectively. According to the 2021 World Health Organization global air quality guidelines and global exposure analyses, approximately 7.3 billion people, representing about 90–94% of the worldwide population live in areas where annual PM2.5 and PM10 concentrations exceed the recommended limits of 5 and 15 µg/m³, respectively [1,2]. Prolonged exposure to PM substantially increases respiratory and cardiovascular mortality and morbidity, including asthma exacerbations, chronic obstructive pulmonary disease (COPD), pneumonia, lung cancer, hypertension, stroke, myocardial infarction, and heart failure [3]. Additional systemic health effects include adverse birth outcomes, neurodevelopmental deficits, metabolic disorders, and immune dysfunction driven by oxidative stress and inflammation, and impaired skin health [4–7].

These particles act as carriers for the adhesion of various detrimental substances, including polycyclic aromatic hydrocarbons (PAHs), heavy metals, organic compounds, and endotoxins, which pose major health risks [5–9]. Air pollution is also known to have detrimental effects on the skin, which is constantly exposed to environmental pollutants [6]. Among these, fine particulate matter (PM2.5) can compromise skin structure and function, contributing to premature aging, inflammatory skin disorders, and barrier dysfunction-related conditions, raising significant concerns for overall skin health [10,11]. Importantly, population-based studies have associated airborne particulate matter exposure to clinical manifestations of extrinsic skin aging, including increased wrinkles and pigmentary changes, supporting PM2.5 as an environmental redox stressor that promoted premature skin aging beyond inflammation alone [11–13].

Oxidative stress and inflammation play a crucial role in PM2.5-induced skin damage, alongside multiple mechanisms including autophagy, apoptosis, DNA damage, mitochondrial dysfunction, skin barrier impairment, and acceleration of the aging process, primarily affecting keratinocytes and dermal fibroblasts [14,15]. At the cellular signaling level, PM2.5-induced reactive oxygen species (ROS) overproduction acts as the initial trigger by oxidatively modifying redox-sensitive proteins, which then activate specific kinases. This ROS-mediated activation stimulates lipid peroxidation, nicotinamide adenine dinucleotide phosphate (NADPH) oxidase, and mitochondria-dependent pathways. These pathways enhance phosphorylation of mitogen-activated protein kinases (MAPKs), namely extracellular signal-regulated kinase (ERK), c-Jun N-terminal kinase (JNK), and p38. Phosphorylated MAPKs, in turn, promote nuclear factor-kappa B (NF-κB) translocation and increase transcriptional activity. This cascade amplifies inflammatory cytokine production, drives transcriptional reprogramming, induces cellular stress responses, and ultimately results in skin barrier dysfunction and premature skin aging [8,14–17]. Several signaling pathways have been implicated in mediating the effects of PM2.5 exposure on skin injury, including the aryl hydrocarbon receptor (AhR), activator protein-1 (AP-1), NF-κB, MAPK, and nuclear factor erythroid 2–related factor 2 (Nrf2) pathways. Targeting key redox and inflammatory signaling pathways may help mitigate skin damage, prevent premature aging, and promote wound healing by restoring cellular homeostasis, thereby preserving skin function and structural integrity [17]. Epidermal keratinocytes, as the primary cellular interface for PM2.5 exposure, detect PM through AhR receptors. This detection initiates ROS and cytokine production, subsequently downregulating key barrier proteins such as filaggrin and loricrin, as well as altering lipid composition. These changes compromise skin barrier integrity and initiate inflammatory responses. Meanwhile, dermal fibroblasts respond to chronic oxidative stress and inflammatory signaling pathways by upregulating matrix metalloproteinase (MMP) expression, reducing collagen synthesis, and undergoing senescence, accelerating extracellular matrix (ECM) degradation and skin aging [10,16,18–20]. Paracrine signaling connects keratinocyte barrier dysfunction to fibroblast-mediated remodeling and photoaging [21]. Additionally, PM2.5-induced endoplasmic reticulum stress and mitochondrial dysfunction promote apoptosis in both cell types [15,22].

Botanicals and their bioactive phytochemicals, found in natural products, have attracted considerable attention as promising therapeutic and preventive strategies for PM2.5-induced skin injury, owing to their potent antioxidant and anti-inflammatory properties. This review addresses the pressing concern of PM2.5-induced skin injury, an underexplored yet increasingly relevant issue in environmental pharmacology. As global levels of air pollution, particularly PM2.5, continue to rise, its impact on skin health has emerged as an urgent public health challenge. While the harmful effects of PM2.5 on respiratory health are well established, its cutaneous effects remain insufficiently investigated, despite clear evidence linking PM2.5 exposure to oxidative stress, inflammation, premature aging, and disruption of the skin barrier. PM2.5 directly interacts with epidermal keratinocytes and dermal fibroblasts, disrupting key signaling pathways that drive oxidative stress, inflammation, cellular dysfunction, and ultimately tissue damage. This review bridges the knowledge gap by integrating PM2.5-driven redox and inflammatory pathways (AhR, NF-κB, AP-1, MAPK, Nrf2) with mechanistic evidence of botanical phytochemicals that specifically modulate these pathways. Unlike previous reviews focused on general air pollution toxicology or antioxidant skincare, we elucidate the mechanisms of PM2.5-induced oxidative stress, redox imbalance, apoptosis, and autophagy disruption, and highlight the therapeutic potential of botanicals, phytochemicals, and natural products. These integrated insights may inform the development of mechanism-based anti-pollution cosmeceuticals, therapeutic strategies, and preventive approaches to reduce PM2.5-associated skin damage and premature aging.

## 2. PM2.5-induced skin injury and premature aging

### 2.1. PM2.5-mediated oxidative stress and disrupted redox signaling

Oxidative stress is defined as a state of imbalance between the production of ROS and the capacity of the body’s antioxidant defense systems to detoxify these reactive intermediates or repair the resulting damage [23]. ROS, including hydrogen peroxide (H_2_O_2_), superoxide anion (O_2_^•−^), and hydroxyl radical (^•^OH), generated upon PM2.5 exposure can directly damage cellular biomolecules, including proteins, lipids, and DNA. This damage occurs primarily through oxidative modifications, thereby disrupting cellular structure and function. In addition to causing direct oxidative damage, ROS also act as important second messengers that disrupt cellular redox signaling, ultimately driving oxidative stress responses and activating inflammatory pathways [24]. PM2.5 matter contains redox-active transition metals such as iron (Fe²^+^) and copper (Cu²^+^) as well as organic compounds including PAHs and quinones [25]. These substances can readily penetrate the skin and airway epithelial barriers through two main mechanisms: direct physical penetration via adherence to the stratum corneum (SC) through trans-epidermal diffusion and follicular routes. Short-term and sub-chronic exposure (4–8 weeks) disrupts epithelial barriers by degrading cell tight junction (TJ) proteins such as ZO-1, occludin, claudin-1, and E-cadherin, which are essential for maintaining barrier integrity in skin and airways. Indirectly, PM activates pattern-recognition and xenobiotic receptors on keratinocytes and airway epithelial cells and triggers ROS overproduction, oxidative stress, inflammation, and further TJ degradation, increasing permeability and disease susceptibility [8,18,26–28]. PM2.5-induced oxidative stress is partially attributed to Fenton-type reactions, in which Fe²^+^ reduces H₂O₂ to generate highly reactive ^•^OH, thereby amplifying intracellular ROS levels [24]. Moreover, PAHs can be metabolically converted into quinones. Redox-cycling quinones exert their toxic effects by undergoing one-electron reductions to form semiquinone radicals, which react with molecular oxygen to regenerate the parent quinone and produce ROS such as superoxide anions (O₂^•−^) and hydrogen peroxide (H₂O₂). These quinones engage in redox cycling, producing ROS and promoting oxidative damage in cells [29].

PM2.5 particles have been shown to contribute to oxidative stress through various mechanisms, including direct cellular interactions, activation of inflammatory responses, interactions between PAHs and cellular components (e.g. receptors and biomolecules), and impaired antioxidant defenses in multiple cell types, such as bronchial epithelial cells, skin cells, neuronal cells, and immune cells [14,30–32]. Although this review primarily focuses on skin biology, evidence from bronchial (BEAS-2B) and neuronal (SH-SY5Y) models is included to illustrate that PM2.5-induced oxidative stress and redox dysregulation represent conserved mechanisms across different barrier and non-barrier tissues. Exposure to PM2.5 in BEAS-2B human bronchial epithelial cells significantly elevated ROS levels, while reducing the activities of antioxidant enzymes catalase (CAT), superoxide dismutase (SOD), and glutathione Peroxidase (GSH-Px) via a combination of Nrf2-dependent antioxidant dysregulation, oxidative post-translational enzyme inactivation, and increased functional consumption under sustained ROS stress [23,33–35]. In SH-SY5Y cells, PM2.5 induced neurotoxicity by increasing NADPH oxidase (NOX)-driven mitochondrial ROS, triggering apoptosis, highlighting NOX4 as a potential therapeutic target [36]. PM2.5 exposure elevated the expression of pro-inflammatory cytokines, including granulocyte-macrophage colony-stimulating factor (GM-CSF), interleukin-6 (IL-6), interleukin-1β (IL-1β), and tumor necrosis factor-alpha (TNF-α). Importantly, this cross-tissue conservation validates the translational relevance of bronchial and neuronal findings to skin barrier disruption via shared molecular pathways involving ROS overproduction, antioxidant depletion, and mitochondrial impairment.

Additionally, PM2.5 amplified lipopolysaccharide (LPS)-induced M1 macrophage polarization without affecting cell viability [37]. The skin functions serve as the primary barrier exposed to environmental pollutants, including PM2.5, making it a major target of oxidative stress. Findings from both *in vitro* and *in vivo* studies have shown that PM2.5 and its components induce the formation of ROS, particularly O_2_^•−^, generated from various endogenous sources such as the mitochondrial respiratory chain [24,38,39]. This PM-induced oxidative damage occurs in multiple cell types such as immune cells, keratinocytes, melanocytes, sebocytes, and dermal fibroblasts, all of which play critical roles in the pathophysiology of various dermatological conditions [8,15,40–43].

Oxidative damage caused by ROS affects lipids, proteins, and DNA, leading to widespread cellular dysfunction. In lipids, ROS initiate lipid peroxidation by targeting polyunsaturated fatty acids, thereby compromising membrane integrity through changes in fluidity and permeability [15,44]. This process cascade generates toxic aldehydes such as 4-hydroxynonenal (4-HNE), which diffuse across membranes and form adducts with proteins and DNA. While low levels of 4-HNE can act as signaling molecules, high concentrations amplify oxidative stress, inflammation, and cell death, contributing to barrier dysfunction, premature aging, and pigmentary changes in the skin following exposure to PM2.5 [15]. Proteins are particularly vulnerable, with ROS oxidizing sulfur-containing and aromatic amino acid molecules, resulting in conformational changes, misfolding, enzyme inactivation, and aggregation through cross-linking [35,45]. In structural proteins such as collagen and elastin, these oxidative modifications accelerate wrinkling and loss of skin elasticity [46]. DNA is another major target of ROS, which causes strand breaks, base modifications such as 8-hydroxy-2′-deoxyguanosine (8-oxo-dG), and DNA–protein crosslinks that hinder replication and transcription. Under physiological conditions, DNA damage response (DDR) pathways maintain genomic stability and prevent cancer through base excision repair (BER) of oxidative DNA damage (e.g. 8-oxo-dG) and p53-dependent checkpoint [47]. However, chronic exposure to PM2.5can dysregulate these protective mechanisms, leading to persistent DNA damage and impaired repair processes. This results in the activation of ataxia telangiectasia and Rad3-related protein (ATR)/checkpoint kinase 1 (CHK1)/p53, autophagy, and inflammatory cascades, establishing a mutagenic feedback loop that promotes carcinogenesis [48–51]. Therefore, chronic oxidative stress caused by environmental pollutants in skin cells accelerates aging and increases the risk of skin cancer [11,52,53]. PM2.5 exposure generated oxidative damage biomarkers, including protein carbonylation, lipid peroxidation [e.g. malondialdehyde (MDA), 4-HNE, 8-iso-prostaglandin-F_2α_ (8-iso PGF_2α_)], and DNA oxidation (e.g. 8-hydroxyguanine, 8-oxoguanine) in keratinocytes and mouse skin [12,15,44]. PM2.5 can disrupt the lipid-rich components of the SC, particularly targeting squalene that is produced by sebaceous glands. The oxidation of squalene contributes significantly to lipid peroxide formation on the skin surface, serving as a biomarker of pollution-induced skin damage. In SZ95 sebocytes, an immortalized human sebaceous gland cells line, PM2.5 exposure progressively induced intracellular ROS production and lipid peroxidation [40,54]. PM2.5-induced lipid and protein oxidation generates byproducts that can function as potent signaling molecules capable of modulating several physiological and pathophysiological processes [8]. By binding to and modifying critical amino acid residues in proteins, 4-HNE-protein adducts can alter the structure and function of key redox-sensitive transcription factors, such as NF-κB, Nrf2, AP-1, and peroxisome proliferator-activated receptors (PPARs), which regulate the expression of genes involved in inflammation, antioxidant defense, cell proliferation, and metabolic homeostasis [55,56]. Exposure of HaCaT keratinocytes to concentrated air particles (CAPs; 0.1–2.5 μm; 5, 10, and 25 μg/mL) for 24 h resulted in significant cytotoxicity, elevated levels of 4-HNE, and increased release of interleukin-1α (IL-1α). The PM concentrations commonly used in vitro (e.g. 25–50 μg/mL) correspond to acute high-exposure conditions that exceed instantaneous ambient mass concentrations but are widely applied to approximate cumulative particle deposition and to elicit measurable mechanistic responses in cell models. These effects were associated with disruption of Nrf2-dependent transcriptional activation of key antioxidant enzymes, comprising glutathione reductase (GR), glutathione peroxidase (GPx), and NADPH-quinone oxidoreductase 1 (NQO1), thereby contributing to oxidative stress and cytotoxicity in keratinocytes [34]. Moreover, PM2.5 exposure also reduced the activities of SOD and GSH-Px, while increasing MDA levels in both normal (PIG1) and vitiligo (PIG3 V) human melanocyte cells [42].

Furthermore, PM exposure can induce mitochondrial damage, affecting not only adenosine triphosphate (ATP) production but also increasing the generation of ROS in response to cellular stress [39,57]. Within mitochondria, PM disrupts the electron transport chain, causing electron leakage that reacts with oxygen to form O_2_^•−^, which subsequently converted into H_2_O_2_ and ^•^OH. PM exposure also stimulates membrane-bound NADPH oxidases, leading to O_2_^•−^ generation in epithelial and immune cells, thereby fueling inflammatory responses. In addition, PM notably enhances xanthine oxidase activity, which produces O_2_^•−^ and H_2_O_2_ during the conversion of hypoxanthine and xanthine to uric acid [58,59]. These mechanisms establish a positive feedback loop whereby PM-induced ROS activate mitochondria, NADPH oxidases, and xanthine oxidase, resulting in secondary bursts of ROS formation [60]. PM2.5 (50 μg/ml) induces apoptosis in HaCaT cells and dermal fibroblasts through endoplasmic reticulum (ER) stress, mitochondrial damage, and oxidative stress, leading to DNA damage, lipid peroxidation, and protein carbonylation [22]. In addition, urban PM (25 and 50 μg/ml) impairs mitochondrial activity and reduces the levels of key proteins, such as sirtuin-1 (SIRT1) and peroxisome proliferator-activated receptor gamma coactivator-1α (PGC-1α), which are critical for regulating mitochondrial biogenesis and energy metabolism in primary human dermal fibroblasts. This reduction was accompanied by an adaptive response to PM-induced stress, marked by increased Nrf2 activity and expression of antioxidant proteins, including glutathione transferases (GST) and SOD2 [61]. However, prolonged or repeated exposure to PM2.5, the adaptive Nrf2 activation appears insufficient to completely restore redox homeostasis. This deficiency declines or dysregulates the antioxidant defense responses, leading to oxidative damage and the accumulation of aging-related phenotypes in dermal fibroblasts [13,53,61].‏

### 2.2. Core signaling hubs activated by PM2.5 in skin cells

The oxidative stress described above acts as a primary trigger for the activation of several core signaling hubs that integrate PM2.5-derived redox imbalance into downstream inflammatory and tissue-remodeling responses. In skin and other barrier epithelium, PM2.5-induced excessive ROS activate phosphorylation of MAPKs, including extracellular signal-regulated kinase1/2 (ERK1/2), JNK, and p38, as well as NF-κB pathway [53,62,63]. ROS can directly or indirectly modulate upstream kinases and phosphatases, leading to sustained phosphorylation of MAPKs. Consequently, it stimulates the IκB kinase (IKK) complex, promoting IκBα degradation and nuclear translocation of NF-κB [62,63]. This MAPK/NF-κB pathway plays various roles, namely, cell proliferation, survival, apoptosis, and inflammation, which contribute to numerous transcriptions of pro-inflammatory cytokines such as TNF-α, IL-1β, IL-6, interleukin-8 (IL-8), as well as chemokines. NF-κB activation directly regulates cyclooxygenase-2 (COX-2) expression by binding to its promoter region, leading to increased COX-2 transcription during oxidative stress, inflammation, and immune responses in keratinocytes, fibroblasts, and macrophages [37,53,64]. In parallel, MAPKs activation also promotes phosphorylation of transcription factors such as c-Jun and c-Fos, leading to the assembly and activation of AP-1 transcription complexes, which upregulate matrix metalloproteinases (MMPs) (MMP-1, MMP-2, MMP-3, MMP-9) and other gene expression responsible for ECM degradation, inflammatory development, and skin aging [13,41,65]. Additionally, previous research on HaCaT keratinocytes, dermal fibroblasts, and 3D skin models has indicated that PM2.5 and urban particulate matter induce excessive ROS production, which subsequently enhances ERK, JNK, and p38 phosphorylation. This signaling cascade elevates COX-2 expression coupled with prostaglandin E2 (PGE2) synthesis and upregulates MMPs levels with enhanced pro-inflammatory cytokine release. These findings support a model where PM2.5-induced ROS serve as upstream signals, converging on MAPK/NF-κB/COX-2 pathways. These emerge as therapeutic targets for oxidative damage and inflammation, barrier disruption, and premature aging in polluted environments [13,53].

### 2.3. PM2.5-induced dysregulation of apoptosis and autophagy

Apoptosis and autophagy are crucial mechanisms for maintaining cellular homeostasis and ensuring the proper function of skin cells, including keratinocytes, fibroblasts, and melanocytes [66,67]. Autophagy facilitates the lysosomal breakdown of proteins, organelles, microorganisms, and external particles, facilitating the clearance of damaged or obsolete cellular components and proteins. Dysregulation of both autophagy and apoptosis, triggered by external factors such as air pollutants and internal stressors, plays a significant role in accelerating skin aging process [63,68]. Moderate autophagy serves a cytoprotective role; however, prolonged or dysregulated autophagy can develop detrimental effects, ultimately leading to autophagy-associated cell death. Consequently, ROS-mediated autophagy in response to PM2.5 reflects a double-edged mechanism: initially protective against oxidative and apoptotic stress, but this can cause epithelial injury and tissue dysfunction when hyperactivated or sustained status [69,70]. Conceptually, autophagy is initially activated as an adaptive, pro-survival response that facilitates the clearance of damaged organelles and restrains intracellular ROS accumulation during oxidative and ER stress. However, when oxidative and ER stress persist or exceed a critical threshold, sustained autophagic signaling transitions into a maladaptive program that cooperates with apoptotic pathways to promote the elimination of irreversibly damaged cells [66,68,71]. ROS generated by PM2.5 play a critical role in triggering apoptosis through multiple interconnected signaling pathways. In the intrinsic apoptosis pathway, excessive ROS production damages mitochondrial membranes, leading to the loss of membrane potential (ΔΨm) and the release of cytochrome c into the cytosol. Cytochrome c then interacts with apoptotic protease activating factor-1 (Apaf-1) and procaspase-9 to form the apoptosome, which activates caspase-9 and downstream caspase-3, expediting apoptosis execution [72]. Moreover, ROS-mediated activation of the MAPK signaling pathway, including ERK1/2, JNK, p38, further develops to apoptosis. PM2.5-induced ROS triggers MAPK activation, along with ERK/JNK/p38 phosphorylation, acting as a redox-sensitive relay that amplifies mitochondrial and ER stress signals toward caspase-dependent apoptosis. Although transient ERK is classically pro-survival, ERK activation under PM2.5 exposure appears context-dependent and is often associated with sustained oxidative stress and pro-apoptotic signaling in keratinocytes [73]. In HaCaT keratinocytes, PM exposure triggers oxidative damage, inflammation, and apoptosis through ERK and p38 activation, which enhances pro-apoptotic proteins such as B-cell lymphoma 2 (Bcl-2) Associated X: Bax and cleaved caspase-3, and suppresses anti-apoptotic proteins B-cell lymphoma 2 (Bcl-2), thereby promoting mitochondrial outer membrane permeabilization and cytochrome c release. Parallel to this, ROS-induced DNA damage activates the tumor suppressor p53, which upregulates pro-apoptotic proteins such as Bax, and represses anti-apoptotic proteins (e.g. Bcl-2 and Bcl-xL), modulating the balance toward apoptosis in both skin and airway epithelial cells [74]. Remarkably, ROS-driven sustained ERK activation functions as a pro-apoptotic signal by promoting Bax expression and caspase-3 activation, relating to oxidative stress to mitochondrial apoptosis pathways [73]. ROS also contribute to ER stress-induced cell death by disrupting ER homeostasis and triggering the unfolded protein response (UPR), which aims to restore cell homeostasis. However, sustained ER stress activates specific UPR components such as C/EBP homologous protein (CHOP), the transcription factor that regulates pro-apoptotic genes. Together with crosstalk between ER and mitochondria, including calcium release and amplification of mitochondrial dysfunction. This shift drives the transition from adaptive stress signaling to apoptosis [75]. Furthermore, ROS-mediated ER stress induces autophagy, which may exert either cytoprotective or detrimental roles depending on the intensity and duration of the stress stimulus [71]. The UPR engages multiple signaling pathways that contribute to autophagy induction. At moderate ROS levels, this ER stress-induced autophagy acts as a cytoprotective mechanism by facilitating the clearance of damaged proteins and organelles, thereby mitigating oxidative burden and preventing apoptosis. However, chronic PM2.5 exposure can shift autophagy from protective to maladaptive response. Sustained activation leads to overexpression of Beclin-1, excessive accumulation of microtubule-associated protein 1 light chain 3-II (LC3-II), and persistent degradation of essential organelles, ultimately resulting in autophagy-associated cell death [76]. Exposure to PM2.5 has been demonstrated to induce autophagy in epithelial cells through mechanisms driven by excessive ROS generation [77]. In human HaCaT keratinocytes, Exposure to PM2.5 enhances autophagosome formation and upregulates key autophagy protein markers such as Beclin-1 and Microtubule-associated protein 1 light chain 3 beta-II (LC3B-II). Importantly, treatment with the antioxidant N-acetyl cysteine (NAC) reverses these changes, highlighting the critical role of ROS in modulating the autophagic response [15]. PM2.5 induces apoptosis in keratinocytes and mouse skin by activating the MAPK signaling pathway, specifically through the phosphorylation of ERK, JNK, and p38 MAPKs [73], and by modulating the oxidative stress-ER stress-autophagy-apoptosis signaling axis [63,71]. Additionally, oxidative stress triggered by PM2.5 led to intracellular Ca^2+^ accumulation, mitochondrial dysfunction, ER stress, ultimately resulting in apoptotic cell death and tissue injury in keratinocytes and mouse skin [15,78–80]. Previous mechanistic investigations have primarily focused on acute exposure durations, ranging from several hours to 1–2 days, to elucidate early stress responses, such as ROS generation, ER stress, and caspase activation. On the other hand, chronic or repeated PM2.5 exposure is more likely to induce long-term maladaptive remodeling, characterized by persistent autophagy, cellular senescence, and ECM degradation in dermal fibroblasts [41,61]. Differentiating between these temporal phases is crucial for accurately interpreting experimental data in actual pollution scenarios. Collectively, these mechanisms illustrate how PM2.5-induced ROS orchestrates apoptosis through a complex network involving mitochondrial dysfunction, MAPK signaling, p53 induction, and ER stress, ultimately contributing to epithelial injury and inflammation.

### 2.4. PM2.5-induced AhR signaling pathway

Air pollutants, including PM2.5, have been shown to compromise both epidermal and dermal integrity, as well as impair skin barrier function, thereby contributing to the pathogenesis of various dermatological conditions such as atopic dermatitis, acne, psoriasis, and premature aging [18]. These effects are mediated, in part, through the modulation of key signaling pathways, including AhR and NF-κB [81–84]. In addition to its canonical role in xenobiotic metabolism, the AhR serves as a critical upstream receptor that connects PM2.5-associated ligands and ROS to the activation of MAPK/NF-κB/AP-1 signaling network. This signaling pathway initiates and amplifies pro-inflammatory responses in keratinocytes, fibroblasts, and immune cells [18,19,81,82,84]. The AhR, a ligand-activated transcription factor expressed in various skin cell types, can bind to a wide range of endogenous and exogenous ligands, including PAHs, components of cigarette smoke, benzo[a]pyrene (BaP), and dioxins. These high-affinity ligands activate the canonical AhR signaling pathway in keratinocytes, melanocytes, fibroblasts, and immune cells, resulting in context-dependent outcomes that range from the physiological regulation of differentiation and barrier function to pathological effects such as dysregulated proliferation, dyspigmentation, and, under sustained or high-intensity exposure conditions, apoptotic cell death and inflammation [83,85–87]. Beyond direct transcriptional responses, AhR activation in keratinocytes can influence biogenesis and cargo loading of exosomes, thereby distributing cytokines, chemokines, and microRNAs that propagate inflammatory signaling to neighboring cells. Exosomes derived from BaP-stimulated human keratinocytes were found to promote the expression of psoriatic cytokines and chemokines, including TNF-α, IL-1β, IL-6, IL-8, C-X-C motif chemokine ligand 1 (CXCL1), and C-X-C motif chemokine ligand 5 (CXCL5), in recipient human keratinocytes [86]. Furthermore, AhR functions as a sensor of environmental-skin interactions, playing a critical role in various physiological functions and phenotypic responses to the environmental changes [87]. Upon activation, AhR is transported to the nucleus, where it forms a dimer with the AhR nuclear transporter (ARNT). This AhR/ARNT protein complex then attaches to the xenobiotic-responsive element (XRE) in the promoter regions of DNA, initiating the transcription of AhR-responsive genes, such as the enzyme cytochrome P450 1A1 (CYP1A1) [88,89]. CYP1A1 plays a critical role in the metabolism of PAHs by facilitating the transformation of PAHs into active intermediates, leading to the production of ROS and mutagenic metabolites. These by-products are highly reactive and can cause significant damage to proteins and DNA, resulting in various negative outcomes for skin health, including premature aging and increased risk of skin cancer. In particular, the crosstalk between AhR andNrf2 under PM2.5 exposure appears to be context dependent. The AhR-driven induction of xenobiotic-metabolizing enzymes (e.g. CYP1A1) can increase the burden of ROS and potentially impair antioxidant defenses, whereas in certain ligand contexts or under pharmacological modulation of AhR, the signaling upstream modulate the Nrf2/antioxidant response element (ARE) pathway as a compensatory antioxidant response [81,88–90]. Treatment of human skin tissue *ex vivo* with PM2.5 was observed to induce the skin barrier dysfunction and inflammation via upregulating AhR pathway in association with the secretion of IL-6 and interleukin-36 gamma (IL-36G) [19]. A previous study using cultured primary keratinocytes and mouse skin models demonstrated that activation of the AhR by PM2.5 induces TNF-α expression, which contributes to the downregulation of the epidermal barrier protein filaggrin [18]. Additionally, phase 3 clinical trials of tapinarof, a topical AhR-modulating agent, have demonstrated clinical efficacy in attenuating psoriasis and atopic dermatitis. Selective AhR modulation by tapinarof restores epidermal barrier proteins, reduces pro-inflammatory cytokine expression, and enhances Nrf2-dependent antioxidant defenses, thereby demonstrating how targeted AhR ligands can be utilized for skin disease treatment [19,87–89,91].

### 2.5. PM-induced activation of inflammatory signaling pathways in skin barrier dysfunction

Building on the upstream activation of AhR described in Section 2.4, PM2.5-mediated inflammation involves a complex interplay between AhR and redox signaling pathways, in which AhR-driven transcriptional changes and ROS production converge on MAPK (ERK/JNK/p38), NF-κB, and AP-1 pathways to sustain cutaneous inflammatory responses [19,62,81]. AhR activation modulates NF-κB activity through indirect mechanisms involving ROS generation rather than direct interactions with NF-κB subunits [92]. Mechanistically, AhR can modulate inflammatory signaling both indirectly, by inducing CYP-mediated ROS and lipid peroxidation products that activate NF-κB, and directly, through protein–protein interactions between AhR and NF-κB subunits that influence nuclear transcriptional complexes. In certain cellular contexts, AhR also acts as a negative regulator of NF-κB signaling via modulation of IKKα/β and IκBα phosphorylation, thereby suppressing NF-κB activation and inflammatory responses. Moreover, AhR promoter regions contain NF-κB binding sites, establishing a feedback loop that links inflammation to AhR expression through ROS- and NF-κB-dependent mechanisms [53,81,92–94]. This crosstalk leads to the upregulation of MAPK signaling and subsequent activation of transcription factors NF-κB and AP-1, which promote the expression of pro-inflammatory cytokines such as TNF-α, IL-1β, IL-6, and IL-8 in human keratinocytes and fibroblasts. Exposure to PM2.5 initiates the overproduction of ROS through pathways involving mitochondrial dysfunction, NADPH oxidases activation, and interactions with redox-active metals. These ROS serve as upstream activators of the NF-κB signaling cascade by stimulating the IKK complex, which phosphorylates and degrades IκBα, thereby releasing NF-κB to translocate into the nucleus and promote transcription of pro-inflammatory cytokines [62]. Studies have consistently identified NF-κB as a central mediator in PM-induced inflammation, connecting oxidative stress to downstream cytokine release in skin and other barrier tissues [62,79,90]. Thus, NF-κB is a key transcription factor that integrates PM2.5-induced oxidative stress via AhR/ROS activation, sustaining inflammatory signaling in skin barrier tissues. This MAPK/NF-κB/AP-1 cascade promotes pro-inflammatory cytokine production (TNF-α, IL-1β, IL-6, IL-8), impairing skin barrier integrity and contributing to the development of disorders such as psoriasis and atopic dermatitis [62,64,79,81,95,96]. Given the critical roles of cholesterol and squalene as crucial indicators of the skin barrier integrity in the epidermis of atopic dermatitis, transcriptome analysis revealed that PM2.5 (50 μg/mL) may compromise the skin barrier by disturbing cholesterol homeostasis, indicated by a transient increase in cholesterol and a simultaneous decrease in squalene, accompanied by upregulation of inflammatory and oxidative stress responses in human primary keratinocytes [64,95–97]. Disruption of cholesterol and squalene in the SC compromises skin barrier integrity. This enhanced penetration facilitates environmental antigens and irritants, thereby amplifying keratinocyte-derived cytokine and inflammatory signaling pathways such as NF-κB and MAPK under PM2.5 exposure. A previous study using cultured HaCaT cells and *in vivo* mouse model, urban PM was demonstrated to downregulate filaggrin involved in the impairment of skin barrier function through upregulating ERK1/2, p38 MAPK/NF-κB, and JNK/AP-1, along with increased COX-2 expression and PGE2 production [20]. PGE2, the major COX-2-derived prostanoid in skin, promotes chronic inflammation and skin barrier dysfunction by downregulating filaggrin in HaCaT cells through COX-2/PGE2 pathway. This impaired barrier function enhances skin permeability and inflammatory responses [20]. Additionally, PGE2 also modulates T helper 2 (Th2)-skewed immune responses, causing inflammatory exacerbation and keratinocyte proliferation in atopic skin. E-prostanoid receptor 2 (EP2) and EP4 receptor activation via PGE2 also induces vascular hyperpermeability, which sustains vasodilation and erythema on mouse skin [17,20,64,98]. In addition, treatment of HaCaT cells with PM2.5 (up to 100 μg/mL) upregulated skin barrier-related proteins, such as filaggrin, involucrin, and loricrin, in association with increased release of TNF-α, IL-1α, and IL-8 [99].

### 2.6. PM2.5-induced upregulation of MMPs and reduction of collagen in premature skin aging

**Figure 1.**
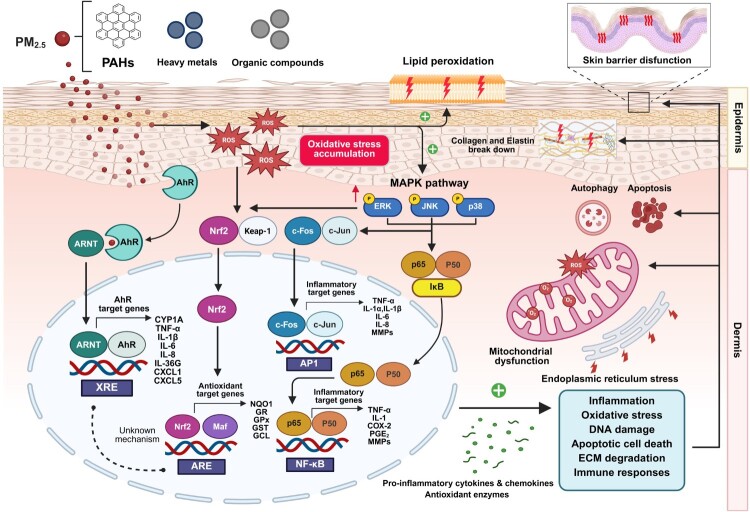
Mechanisms of PM2.5-induced skin injury through oxidative stress and inflammation. PM2.5 exposure, containing PAHs, heavy metals, and organic compounds, initiates skin damage by generating ROS and inducing lipid peroxidation, resulting in oxidative stress. This accumulation of ROS activates the AhR, which subsequently upregulates the transcription of pro-inflammatory genes and cytokines such as COX-2, TNF-α, IL-1 and IL-6. In parallel, MAPK signaling pathway is also activated. Together, oxidative stress and AhR signaling enhance inflammatory and degradative responses through the activation of NF-κB and AP-1, leading to the breakdown of collagen and elastin. PM2.5 exposure also downregulates the activity of Nrf2, a key transcription factor responsible for regulating the antioxidant defense system. These detrimental effects of PM2.5 ultimately result in sustained inflammation, oxidative damage, DNA damage, apoptosis, ECM degradation, and allergic immune responses. Collectively, these processes contribute to overall skin injury, including premature aging and impaired barrier function. Abbreviations: PAHs: polycyclic aromatic hydrocarbons; ROS: reactive oxygen species; AhR: aryl hydrocarbon receptor; ARNT: AhR nuclear transporter; COX-2: cyclooxygenase-2; TNF-α: tumor necrosis factor- α; IL-: interleukin-1; IL-6: interleukin-6; MAPKs: mitogen-activated protein kinases; ERK: extracellular signal-regulated kinase; JNK: c-Jun N-terminal kinase; p38: p38 mitogen-activated protein kinase; NF-κB: nuclear factor kappa B; p65: RelA nuclear factor-κB homodimer; p50: nuclear factor-κB 1 homodimers; AP-1: activator protein 1; Nrf2: nuclear factor erythroid 2-related factor 2; Maf: transcription factor Maf; ARE: antioxidant response element; IκB: IκB kinase; MMPs: Matrix Metalloproteinases; ECM: extracellular matrix; XRE: xenobiotic-responsive element

Fibroblasts in the dermal layer play a pivotal role in synthesizing ECM proteins, such as collagen and elastin, which provide structural support and maintain skin elasticity. Environmental stressors that disrupt the balance of MMPs production or activity, including MMP-1 (collagenase-1), MMP-2 (gelatinase A), MMP-3 (stromelysin-1), and MMP-9 (gelatinase B), can collectively accelerate the degradation of ECM components. This degradation compromises skin integrity and contributes to visible signs of aging, such as reduced elasticity and wrinkle formation [52,100]. ROS and inflammatory signaling pathways play a critical role in PM-induced upregulation of MMPs in dermal fibroblasts. Urban PM (up to 50 μg/mL) has been shown to upregulate the expression of MMP-1 and MMP-3, a response associated with the induction of transforming growth factor-beta (TGF-β) through activation of AhR and increased ROS formation in dermal fibroblasts [97,101]. TGF-β is a multifunctional cytokine that plays a critical role in regulating cell growth, differentiation, and ECM homeostasis. The TGF-β pathway maintains skin structural integrity and skin repair by regulating collagen synthesis in fibroblasts via MMPs [46,102]. PM2.5 and PM10 can dysregulate both canonical and non-canonical TGF-β signaling pathways that converge on fibrosis, inflammation, epithelial–mesenchymal transition (EMT) and downstream changes in MMP-mediated collagen degradation in various tissues such as lung, skin and hepatic tissues [41,103–106]. In canonical signaling, TGF-β1 binds type II/I receptors (TβRII/TβRI) and activates Smad2/3 phosphorylation, forming a complex with Smad4 to develop fibrosis by suppressing MMPs and enhancing collagen deposition [46,102]. However, PM2.5-induced chronic oxidative stress switches TGF-β toward non-canonical MAPK (ERK, JNK, p38) pathways in skin fibroblasts, upregulating MMP-1, −3, and −9 and accelerating ECM degradation and skin aging [41,46,52,107,108]. ROS generated by PM2.5 contribute to the upregulation of MMP-1, MMP-2, and MMP-9 via activation of the MAPK/AP-1 signaling pathway in HaCaT cells [65]. Furthermore, PM2.5-mediated inflammatory responses can disrupt the ECM by inhibiting the synthesis of collagen and elastin, while concurrently promoting the expression of MMPs [100]. Additionally, PM2.5 and urban particulate extracts have been reported to directly suppress collagen Type I Alpha 1 Chain (COL1A1) and collagen type III alpha 1 chain (COL3A1) transcription, inhibit procollagen synthesis, and induce senescence-like phenotypes in dermal fibroblasts [13,41,61]. These effects impair ECM replenishment while enhancing matrix degradation, establishing a direct mechanistic link between PM exposure and clinically observed premature skin aging [11–13]. PM also enhances the expression of pro-inflammatory cytokines IL-1α and IL-1β in keratinocytes through activation of the p38 MAPK pathway, which is further associated with increased MMP-1 and COX-2 expression in dermal fibroblasts [109]. PM2.5 was found to induce activation of JNK/NF-κB pathways, which mediated IL-6 and TNF-α production, in association with upregulating MMP-1 expression in cultured dermal fibroblasts and a 3D-skin model [14]. Treatment of dermal fibroblasts with conditioned media derived from PM-treated HaCaT cells increased intracellular ROS, pro-inflammatory cytokines, and MMPs (MMP-1 and MMP-2), together with enhanced collagenase and elastase activities, demonstrating that PM-exposed keratinocytes can propagate oxidative and inflammatory signaling to dermal fibroblasts via paracrine mechanisms, thereby amplifying dermal remodeling responses beyond direct particle exposure [21]. These findings highlight the critical role of keratinocyte-fibroblast paracrine signaling in PM-induced dermal remodeling. A schematic overview of the redox and inflammatory signaling pathways induced by PM2.5 is presented in Figure 1.

## 3. Development of botanicals and phytochemicals as promising protective agents for PM2.5-induced skin injury

Natural products, including botanicals, phytochemicals and nutraceuticals derived from plants, animals, and microorganisms, play significant roles in therapeutic approaches, wellness, and health promotion. Currently, the application of natural compounds in protecting against PM 2.5-induced skin injury has significantly gained attention in recent research, particularly air pollution-related skin damage [110]. Focusing on the pharmacological aspect, botanicals and their bioactive phytochemicals serve complementary and interconnected roles, bridging traditional medicine with modern drug discovery. Botanicals are complex mixtures of plant-derived components, containing phytochemicals and others that often act synergistically to produce therapeutic purposes, whereas isolated phytochemicals allow the study of targeted molecular mechanisms with greater precision. However, botanical extracts significantly present limitations, including batch-to-batch variability arising from differences in plant source, harvesting conditions, and extraction methods. These standardization and quality control assurance challenges compromise reproducibility and cross-study comparability, preventing consistent therapeutic outcomes across studies [110–112]. The dual approach, investigating whole botanical extracts for their synergistic, multi-target actions while also characterizing the molecular pharmacology of purified phytochemicals, has significantly contributed to therapeutic innovation [111,112]. Phytochemicals, non-nutritive compounds, are classified as primary and secondary metabolites in plants, based on their metabolic processes. Numerous plant secondary metabolites are responsible for plant-environment interactions and stress defense mechanisms. These metabolites are typically classified as low-molecular-weight molecules produced during various stages of plant metabolism. Moreover, these compounds are broadly distributed across many dietary and medicinal sources, including vegetables, fruits, legumes, herbs, aromatic plants, and diverse plant parts such as leaves, flowers, and roots [113].

As described in our previous review, polyphenols can be categorized into phenolic acids, flavonoids, stilbenes, and lignans based on their structural characteristics and carbon content [114]. Many phytochemicals not only directly scavenge ROS, but also modulate key signaling pathways, particularly NF-κB, MAPK, and Nrf2, thereby delivering a range of beneficial biological effects, including antioxidant, anti-inflammatory, immunomodulatory, chemopreventive, and cytoprotective actions relevant to chronic diseases and environmental stress-related disorders [115–117].

**Figure 2.**
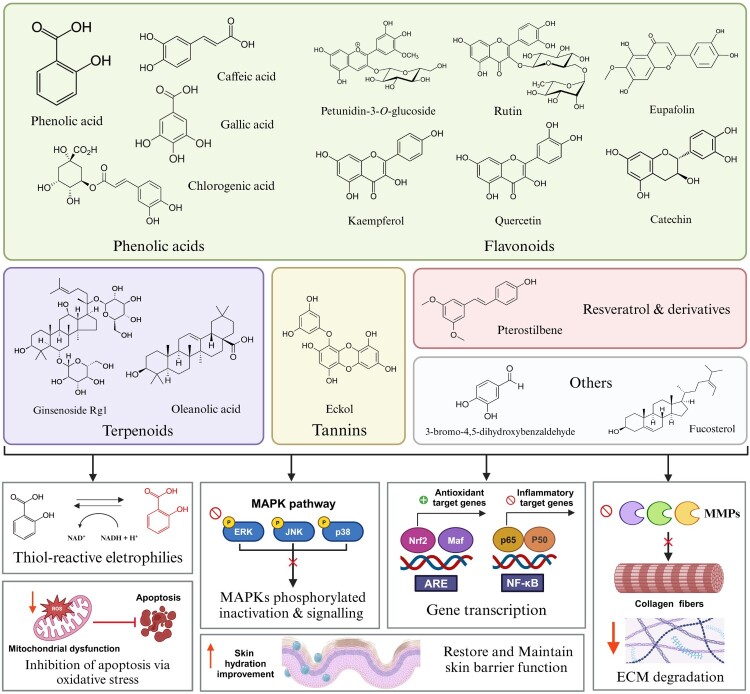
Bioactive phytochemicals and their mechanisms of action against PM2.5-induced skin damage. Various phytochemicals exert protective effects against oxidative damage, apoptosis, skin aging and skin barrier dysfunction through multiple mechanisms including scavenging ROS, restoring mitochondrial function, inactivating MAPKs (ERK, JNK, p38), activating Nrf2/ARE pathway to enhance antioxidant defenses, suppressing NF-κB-mediated inflammatory responses and inhibiting MMPs activity to prevent collagen and ECM degradation. Abbreviations: ROS: reactive oxygen species; MAPK: mitogen-activated protein Kinase; Nrf2: nuclear erythroid 2-related factor 2; ARE: antioxidant response element; NF-κB: nuclear factor kappa B; p65: RelA nuclear factor-κB homodimer; p50: nuclear factor-κB 1 homodimers; MMPs: Matrix metalloproteinases; ECM: extracellular matrix.

This review focuses on the role of botanicals and bioactive phytochemicals in protecting against PM-induced skin damage through multiple biological actions, particularly their antioxidant, anti-inflammatory, and cytoprotective properties (Figure 2). As discussed in the previous sections, the cell’s ability to counteract oxidative damage relies on the activation of the Nrf2/ARE pathway, which is essential for maintaining cellular homeostasis in response to chemical and oxidative stress, particularly in skin cells such as keratinocytes, fibroblasts, and melanocytes [118]. Exposure to PM2.5 has been associated with impaired antioxidant capacity across multiple organs, including the heart, kidney, liver, and lungs, by reducing intracellular glutathione (GSH) levels and the activity of key antioxidant enzymes such as SOD and GPx [119,120]. Nrf2 is a master redox-sensitive transcription factor that regulates the expression of antioxidant and cytoprotective genes, playing a vital role in the skin’s defense against environmental stressors, including PM2.5. Nrf2 activity is tightly regulated by its cytoplasmic repressor Kelch-like ECH-associated protein 1 (Keap1) and the proteasomal degradation system. Under basal conditions, Keap1 binds to Nrf2 and targets it for ubiquitination via the Keap1-Cul3 E3 ligase complex, leading to its degradation. However, in response to ROS generated by environmental stressors or electrophilic agents, specific cysteine residues on Keap1, particularly cysteine 151 (C151), undergo oxidative modification. This induces a conformational change in Keap1, disrupting the Keap1-Nrf2 complex and allowing Nrf2 to escape degradation. Stabilized Nrf2 translocates to the nucleus, where it binds to the ARE, a cis-regulatory enhancer located in the promoter regions of phase II detoxifying and antioxidant genes, including glutamate-cysteine ligase (GCL), GST, and NQO1 [121–123]. Mechanistically, phytochemicals can function as thiol-reactive electrophiles by covalently modifying cysteine residues, most notably C151, on Keap1, thereby disrupting the Keap1-Cul3 E3 ligase complex and stabilizing Nrf2 [124]. The accumulated Nrf2 enhances the transcription of antioxidant and cytoprotective genes, strengthening the cellular defense response against environmental insults such as PM2.5 and UV radiation [125]. Nrf2 activators, including the isothiocyanate sulforaphane (SFN), the alkylating agent iodoacetamide and tert-butylhydroquinone (tBHQ), modify C151 residue in Keap1, thereby enhancing Nrf2’s nuclear accumulation [116]. Notably, these compounds have also been observed to protect against PM-induced lung and skin epithelial injury via activating Nrf2 signaling [90,126].

As discussed in Sections 2.1–2.4, PM2.5-induced AhR activation and subsequent ROS-driven MAPK/AP-1 and NF-κB cascades represent the central inflammatory response in skin cells [90]. In therapeutic perspective, phytochemicals simultaneously target and modulate MAPK/AP-1 signaling through ERK/JNK/p38 inhibition, which disrupts PM2.5-induced non-canonical TGF-β signaling in skin fibroblasts, and suppress NF-κB activation by inhibiting IKK phosphorylation, preventing IκBα degradation, and blocking p65 nuclear translocation. This modulation effectively suppresses MMPs induction and pro-inflammatory cytokine release and TGF-β/Smad signaling hyperactivation [126–130]. Concurrently, phytochemicals enhance Nrf2-mediated cytoprotection through HO-1/NQO1 upregulation, thereby counteracting PM2.5-induced skin injury, barrier dysfunction, and premature aging processes such as collagen degradation, dermal barrier disruption, and ECM remodeling [131–133].

Beyond these phytochemicals and natural products, Traditional Chinese Medicines (TCMs) and bioactive compounds such as Salvianolic acid B isolated from *Salvia miltiorrhiza* and polyherbal formulations including Bazi Bushen capsule provides complementary therapeutic potential to mitigate oxidative stress and skin aging in *in vitro/in vivo* models. Salvianolic acid B demonstrates potent antioxidant and metal-chelating properties, effectively suppresses MAPK/NF-κB signaling, and reduces MMP-1/MMP-3 expression while preserving collagen in oxidative stress or UV-irradiated skin models [134,135]. Similarly, Bazi Bushen capsule improves mitochondrial function and enhance collagen and elastin content through Nrf2, SIRT1, and TGF-β/Smad pathways [136]. Although PM2.5-specific studies remain limited, these TCMs demonstrate considerable promise for further mechanistic and clinical investigations into PM2.5-induced skin damage. In this review focusing on mitigating PM2.5, several *in vitro*, *in vivo*, and clinical studies have demonstrated the protective roles of botanicals and phytochemicals in mitigating PM-induced oxidative damage, inflammation, skin barrier dysfunction, and premature aging. The following sections describe and summarize (Table 1) the accumulated evidence on the pharmacological potential of a range of natural compounds to combat the adverse effects of PM2.5 on the skin.

**Table 1.** The protective roles of botanicals and phytochemicals in mitigating PM-induced oxidative damage, inflammation, skin barrier dysfunction, and premature aging.

Phytochemicals	Active compounds and Sources	Effects	Treatment and study model	Mechanism of action
Flavonoids and phenolic acids	Petunidin-3-O-glucoside (anthocyanin) from black bean extract and phenolics (rutin, catechin and gallic acid) [137]	Anti-oxidative stress Anti-aging	Treatment with gallic acid (100 μM), rutin (100 μM), catechin (100 μM), crude black bean extract (100 μg/mL), crude pinto bean extract (100 μg/mL), phenolic enriched black bean extract (100 μg/mL), and phenolic enriched pinto bean extract (100 μg/mL) for 24 h prior to PM10 (0.1-1000 μg/mL) *In vitro:* human dermal fibroblasts cell lines (Hs27, Hs68)	↓ intracellular ROS ↑ cellular energy homeostasis (adenosine monophosphate activated protein kinase: AMPK)-sirtuin-1 pathway
	*Laminaria japonica* from fermented sea tangle extract [80]	Anti-oxidative stress Anti-apoptosis	Treatment with fermented sea tangle extract (100, 200, 400, 800, and 1,600 μg/mL) for 16 h prior to PM2.5 (50 μg/mL) *In vitro*: keratinocyte HaCaT cells	↓ intracellular Ca^2+^ accumulation and apoptosis ↓ mitochondrial damage
Flavonoids and phenolic acids	Siegesbeckiae Herba extract [138]	Anti-oxidative stress	Treatment with Siegesbeckiae Herba extract (50 μg/mL), absence or presence of PM10 (200 μg/mL) for 48 h *In vitro*: keratinocyte HaCaT cells	↓ ROS and lipid peroxidation ↓ negative regulator of Nrf2 (KEAP1) and ↑ Nrf2 targeted genes (heme oxygenase 1 and NQO1) ↑ antioxidant defense genes (GCLC, GCLM), GSH content
	Eupafolin from *Phyla nodiflora* [140]	Anti-oxidative stress Anti-inflammation	Treatment with eupafolin nanoparticle delivery system (ENDS) (10 µM) for 1 h prior to PM (50 µg/cm^2^) for 1 h *In vitro*: keratinocyte HaCaT cells	↓ ROS production and nicotinamide adenine dinucleotide phosphate oxidase activity (NADPH) oxidase activity ↓ COX-2 expression and PGE2 production through downregulation of MAPK and NF-κB signaling
Flavonoids and phenolic acids	Tart cherry (*Prunus cerasus* L.) extract (chlorogenic acid, quercetin, and kaempferol) [139]	Anti-oxidative stress and Anti-apoptosis	Treatment with tart cherry extract (200 μg/mL) and PM10 (100 μg/mL) for 24 h *In vitro*: keratinocyte HaCaT cells	↓ intracellular ROS ↓ apoptosis-related genes (Bcl-2, Bax, and caspase-3) ↓ ERK and p38 MAPK pathways
Tannins	Green tea extract (GTE) [97]	Anti-oxidative stress Anti-inflammation	Treatment with GTE (0.6%) with or without PM2.5 (50 μg/mL) for 2, 4, 6 days *In vitro*: 3D skin model (epidermis tissue model)	↓ epidermal cholesterol levels, the amount of squalene ↓ genes involved in cytokine-mediated signaling, IL-4 and IL-13 signaling
	Eckol, a phlorotannin isolated from brown seaweed [141]	Anti-apoptosis Anti-oxidative stress Anti-inflammation	Treatment of cells with eckol (30 μM) and/or PM2.5 (50 μg/mL) for 24 h *In vitro*: keratinocyte HaCaT cells	↓ intracellular ROS, lipid peroxidation, protein carbonylation, and DNA damage ↓ mitochondrial dysfunction ↓ apoptosis by decreased cleaved caspase-3 and pro-apoptotic Bax, increased anti-apoptotic Bcl-2 and inactivation of MAPK signaling pathways
Resveratrol and its derivatives	Pterostilbene [142]	Anti-oxidative stress Anti-inflammation Anti-aging Improved skin barrier function	Treatment with Pterostilbene (20 µM) prior to PM (50 µg/cm^2^) *In vitro*: keratinocyte HaCaT cells and CCD-966SK dermal fibroblasts	↓ intracellular ROS ↓ the translocation of AhR into the nucleus and its downstream target gene CYP1A1 ↓ pro-inflammatory mediator COX-2 expression and MAPK phosphorylation ↓ protein levels of MMPs: MMP-1, MMP-2, and MMP-9 ↑ moisturizing protein aquaporin-3 (AQP-3)
Terpenoids: diterpene, triterpene, and sesquiterpene	Oleanolic acid (bioactive compound of *L. lucidum*) [143]	Anti-autophagy Anti-inflammation Anti-aging	Treatment with *Ligustrum lucidum* (20 μg/mL) and oleanolic acid (2.5, 5, 10 μg/mL) for 6 h prior to PM10 (50 μg/mL) *In vitro*: keratinocyte HaCaT cells and dermal fibroblasts	↓ the activation of AhR ↓ autophagy (microtubule-associated protein 1 light chain 3-II (LC3-II) and p62 proteins) ↓ TNF-α mRNA and IL-6 protein ↓ MMP-1 secreted from dermal fibroblasts
Terpenoids: diterpene, triterpene, and sesquiterpene	Ginsenoside Rb1 [22]	Anti-oxidative stress Anti-apoptosis	Treatment with Ginsenoside Rb1 (40 μM) prior to PM2.5 (50 μg/mL) *In vitro*: keratinocyte HaCaT cells and dermal fibroblasts	↓ ROS generation, DNA damage, lipid peroxidation, and protein carbonylation ↓ ER stress-related genes such as protein kinase R-like ER kinase (PERK), inositol-requiring enzyme 1 (IRE1), activating transcription factor (ATF), and CHOP ↓ mitochondrial damage
	*Caulerpa racemosa* (clionasterol rich hexane fraction) [144]	Anti-oxidative stress and Anti-apoptosis	Treatment with clionasterol rich hexane fraction (25, 50, 100, and 200 μg/mL) prior to PM (200 μg/mL) *In vitro*: keratinocyte HaCaT cells Treatment with clionasterol rich hexane fraction (25, 50, 100 μg/mL) for 1 h before PM (200 μg/mL) for 24 h in embryo medium *In vivo*: a zebrafish model	↓ intracellular ROS and mitochondrial ROS production ↓ mitochondria-mediated apoptosis-related proteins (Bax, caspase-3, p53, caspase-9, and cleaved poly (ADP-ribose) polymerase (PARP)) ↓ ROS production, lipid peroxidation, and cell death
Other natural products	*Lycium barbarum* polysaccharide [71]	Anti-oxidative stress Anti-apoptosis Anti-autophagy	Treatment with *Lycium barbarum* polysaccharide (2.5 μg/mL) for 2 h prior to PM2.5 (100 μg/mL) *In vitro*: keratinocyte HaCaT cells	↓ oxidative stress by reduced intracellular ROS and lipid peroxidation (MDA) and increased SOD activity ↓ ER stress-C/EBP homologous protein (CHOP) apoptosis signal and conversion of LC3I to LC3II ↓ apoptosis by inhibiting mitochondrial damage, downregulating pro-apoptotic Bax, and upregulating the anti-apoptotic Bcl-2 protein.
	*Lactobacillus plantarum* MG4221 from the fermented blueberry and black rice extract (FBBBR) [145]	Anti-inflammation and improved skin barrier function	Treatment with FBBBR (100, 250, 500 μg/mL) prior to PM2.5 (500 μg/mL) *In vitro*: keratinocyte HaCaT cells	↓ pro-inflammatory cytokines (IL-1β, IL-6, and IL-8) ↓ NF-κB and MAPK pathways
Other natural products	*Lactobacillus plantarum* MG4221 from the fermented blueberry and black rice extract (FBBBR) [145]	Anti-inflammation and improved skin barrier function	Oral administration of FBBBR extract (10, 50, 100 mg/kg) and then PM2.5/dinitrochlorobenzene (DNCB) (10 mL/kg) *In vivo*: NC/Nga mice (atopic dermatitis mice)	↓ transepidermal water loss (TEWL), skin erythema ↓ serum IgE and T helper 2-associated cytokine ↓ inflammation (p-IκB and NF-κB) ↑ moisturization (FLG and IVL, the protein markers of skin barrier function)
	3-bromo-4,5-dihydroxybenzaldehyde (3-BDB) from Marine algae [146]	Anti-oxidative stress Anti-apoptosis Anti-inflammation Anti-aging	Treatment with 3-BDB (30 μM) for 1 h prior to PM2.5 (50 μg/mL) for 24 h *In vitro*: keratinocyte HaCaT cells Topical administration of 3-BDB (0.3 mM or 3 mM) 30 min before PM2.5 (100 μg/mL) to the dorsal portion of mouse skin for 7 days *In vivo*: HR-1 hairless male mice	↓ intracellular ROS, lipid peroxidation and DNA damage (8-oxoG) ↓ mitochondrial dysfunction, suppressing apoptotic markers (Bax, caspase-9, and caspase-3), and increasing the anti-apoptotic protein Bcl-2 ↓ AP-1/MAPK pathways and pro-inflammatory cytokines (IL-1β and IL-6) ↓ senescence-related proteins (β-galactosidase) ↓ MMPs: MMP-1, MMP-2, and MMP-9
Other natural products	*Sargassum horneri* (Turner) [147]	Anti-oxidative stress Anti-inflammation and improvement of skin barrier function	Treatment with *Sargassum horneri* (31.3–125 μg/mL) prior to fine dust (150 μg/mL) *In vitro*: keratinocyte HaCaT cells	↓ intracellular ROS ↓ pro-inflammatory cytokines (IL-1β, IL-6, IL-8, and TNF-α) ↓ epithelial cytokines (IL-25, IL-33, and thymic stromal lymphopoietin (TSLP)) and chemokines (thymus and activation-regulated chemokine (TARC), macrophage-derived chemokine (MDC), and regulated upon activation normal T cell expressed and secreted (RANTES)) ↓ NF-κB and MAPK pathways
	Fucosterol from Brown algae [21]	Anti-oxidative stress Anti-apoptosis Anti-inflammation Anti-aging	Treatment with fucosterol (12.5, 25, 50 μg/mL) prior to PM (125 μg/mL) *In vitro*: keratinocyte-fibroblast culture system	↓ ROS production and apoptotic body formation ↓ pro-inflammatory cytokines (IL-1β, IL-6, and TNF-α) ↓ collagenase and elastase activity ↓ levels of MMP-1 and MMP-2

### 3.1. Flavonoids and phenolic acids

Flavonoids and phenolic acids demonstrate substantial protective effects against PM-induced skin damage, primarily through antioxidant and anti-inflammatory mechanisms. Petunidin-3-O-glucoside, anthocyanin, and phenolics (rutin, catechin, and gallic acid) against PM10-induced oxidative stress in dermal fibroblasts [137]. Siegesbeckiae herba extract containing chlorogenic acid ameliorated PM10-mediated cytotoxicity and oxidative stress via upregulating Nrf2 target genes, including heme oxygenase 1 (HMOX1) and NQO1 as well as GSH content [138]. Furthermore, Tart cherry extracts, rich in chlorogenic acid, quercetin, and kaempferol, protected against PM10-mediated mitochondrial pathway of apoptosis and oxidative stress in HaCaT cells by reducing ROS formation and downregulating apoptosis-related genes (Bcl-2, Bax, and caspase-3) as well as inhibiting MAPK and NF-κB signaling pathways [139]. Preliminary findings include *Laminaria japonica* (sea tangle) extracts, rich in bioactive polyphenols, amino acids, and polysaccharides, mitigated PM2.5-induced mitochondria-dependent apoptosis via inhibiting ROS formation, DNA damage, lipid peroxidation and protein carbonylation in HaCaT keratinocytes [80]. The phenolic eupafolin protects HaCaT cells from PM-induced inflammation and oxidative stress by modulating the ROS/MAPK and COX2/PGE2 signaling pathways [140]. Overall, while various flavonoids and phenolic acids demonstrate ROS scavenging, Nrf2 activation, and NF-κB/MAPK pathways across multiple preclinical studies, these compounds have not yet been investigated in clinical trials or actual PM exposure, highlighting a key translational gap that future research should address [137–140].

### 3.2. Tannins

Green tea extract (GTE) mitigated PM2.5-induced damage of skin epidermis by restoring epidermal lipid cholesterol homeostasis in association with its anti-inflammatory and antioxidant properties using the 3D skin model [97]. Eckol, a phlorotannin isolated from brown seaweed, demonstrated protective effects against PM2.5-induced apoptosis in HaCaT cells by mitigating mitochondrial dysfunction, reducing oxidative damage to lipids, proteins, and DNA, and downregulating the MAPK signaling pathway [141].

### 3.3. Resveratrol and its derivatives

Pterostilbene, a naturally occurring dimethylated derivative of resveratrol, mitigates PM-induced skin inflammation, aging, and impaired barrier function by downregulating AhR activity and its downstream target gene CYP1A1, as well as suppressing COX-2 expression and MAPK phosphorylation. Additionally, it lowered protein levels of MMP-1, MMP-2, and MMP-9, which are indicative of skin aging, while increasing the moisturizing protein aquaporin-3 (AQP-3) in HaCaT cells and CCD-966SK dermal fibroblasts [142].

### 3.4. Terpenoids: diterpene, triterpene and sesquiterpene

Oleanolic acid, a ubiquitous pentacyclic triterpenoid, is a bioactive compound of L. lucidum, has been demonstrated to suppress PM10 induced p62 and LC-3 I and II proteins involved in autophagy process via inactivation of AhR signaling pathway and reduction of TNF-α mRNA and IL-6 protein in HaCaT cells. Moreover, oleanolic acid was also able to inhibit MMP-1 secreted from dermal fibroblasts pretreated with conditioned media derived from PM10-treated HaCaT cells [143]. Treatment of HaCaT cells and dermal fibroblasts with 40 μM ginsenoside Rb1, a triterpenoid saponin, conferred protection against PM2.5 induced apoptosis. This protective effect was mediated by an attenuation of ER stress, mitochondrial damage, and oxidative damage to DNA, lipids, and proteins [22]. A fraction of *Caulerpa racemosa* containing the triterpenoid clionasterol demonstrated inhibitory effects against PM-induced skin damage in both human keratinocytes and a zebrafish model by suppressing oxidative stress and inhibiting mitochondrial-mediated apoptosis [144].

### 3.5. Other natural products

*Lycium barbarum* polysaccharide showed protective effects against PM2.5-induced apoptosis and autophagy in HaCaT cells in association with suppressing oxidative stress, ER stress-CHOP apoptosis signal and mitochondrial damage [71]. In addition, the fermented blueberry and black rice extract (FBBBR) containing *Lactobacillus plantarum* MG4221 showed protective effects on PM2.5-induced inflammation in HaCaT cells via inactivation of MAPK/NF-kB pathways. In PM2.5/dinitrochlorobenzene (DNCB)-treated NC/Nga mice, oral administration of FBBBR also reduced transepidermal water loss (TEWL) and erythema, scratching behavior in association with reduction of serum immunoglobin E and T helper 2-associated cytokine and upregulation of filaggrin and involucrin in the skin of atopic dermatitis mice [145]. In addition, the marine algae compound 3-bromo-4,5-dihydroxybenzaldehyde (3-BDB) effectively protects against PM2.5-induced skin damage, oxidative stress, and inflammation both *in vitro* and *in vivo*. Furthermore, 3-BDB reverses PM2.5-induced cellular senescence, apoptosis, and mitochondrial dysfunction, in part through the downregulation of the MAPK/AP-1 signaling pathway [146]. The ethanol extract of *Sargassum horneri* (Turner) C. Agardh alleviates fine dust-induced inflammatory responses and skin barrier dysfunction in HaCaT cells [147]. Fucosterol, one of the most abundant sterols in brown algae, reduced PM-induced inflammatory responses, oxidative stress and MMPs in skin cells by downregulating AP-1, NF-κB and MAPK signaling pathways in an integrated keratinocyte-fibroblast culture system [21].

## 4. Clinical evidence of botanicals and phytochemicals as promising protective agents against PM2.5-induced skin toxicity

PM toxicity to the skin has frequently been investigated indirectly by comparing populations living in areas with varying pollution levels [148]. Both topical and oral interventions containing antioxidant compounds or phytochemicals have shown promising effects in both preclinical and clinical settings against pollution-induced skin injury [149]. Topical interventions have provided early proof-of-concept that antioxidant mixtures can blunt pollution-related proinflammatory responses in human skin. In a 4-day single-blinded clinical study (*n* = 15 healthy female volunteers; back test sites) under a real-life multi-pollutant exposure setting, daily application of a serum containing 15% ascorbic acid, 0.5% ferulic acid, and 1% tocopherol mitigated PM- and UV-induced skin damage by maintaining the skin barrier markers (involucrin and loricrin), reducing lipid peroxidation (4-HNE), inflammatory markers (COX-2, NOD-like receptor (NLR) family pyrin domain-containing 1 (NLRP1), AhR and MMP-9), and type 1 collagen degradation in a human clinical trial [150]. *In vitro* and clinical studies have evaluated the effects of Blue Fenugreek Kale Extract (BFKE) on skin aging. *In vitro*, BFKE (0.001–0.01 mg/mL) significantly reduced protein carbonylation in dermal fibroblasts stressed with 0.1 µg/cm^2^ PM and 1 J/cm^2^ UVA. Oral supplementation studies also suggest clinically measurable benefits in polluted environments. In a randomized, double-blind, placebo-controlled trial conducted in Mumbai, India (*n* = 59 volunteers; duration = 56 days), ingestion of Blue Fenugreek Kale Extract (BFKE; 175 mg/capsule, containing flavonoid glycosylates) significantly improved skin hydration and restored barrier function compared with placebo, supporting the feasibility of systemic botanical interventions for pollution-exposed populations [151]. In a randomized, double-blind clinical study conducted in a polluted urban European setting (Milan, Italy), 100 Asian and Caucasian women aged 35–60 years received a polyphenol-enriched dietary supplement based on four standardized plant extracts (*Olea europaea leaf, Lippia citriodora, Rosmarinus officinalis, and Sophora japonica*) for 12 weeks, resulting in reduced oxidative skin damage and significant improvements in skin radiance, moisturization, TEWL, and pigmentation, with measurable benefits observed as early as 2 weeks of supplementation [152,153]. Topical antipollution/antioxidant formulations have also been evaluated in controlled prospective designs. In a single-blind, prospective trial in women living in Rome, Italy (n = 20; 4 weeks), an antioxidant serum containing *Deschampsia antarctica* extract, ferulic acid, and vitamin C improved hydration and reduced markers of pollutant-related skin stress compared with baseline/controls [154]. Barrier-focused strategies that limit pollutant deposition provide an additional clinical approach to mitigating pollution-related skin damage. In a controlled split-face study conducted in a polluted urban setting (*n *= 30 healthy volunteers; 6-h exposure), application of a multicomponent powder (Vitachelox®), composed of grape seed, green tea, and oak wood extracts, significantly reduced the accumulation of polluting ions on the skin surface, while a related topical serum evaluation over four weeks demonstrated improvements in barrier function and oxidative status, including reduced TEWL, increased skin brightness, and decreased lipid peroxidation as reflected by a lower squalene peroxide/squalene ratio [155]. Finally, a monocentre, double-blind, randomized, placebo-controlled crossover trial conducted among outdoor workers in Beijing, China, an environment characterized by persistently high PM2.5 levels demonstrated that oral Pycnogenol® supplementation (2 × 50 mg/day) over 12 weeks (*n* = 76) prevented seasonal declines in skin hydration, reduced TEWL, and improved viscoelastic parameters, indicating systemic protective effects under chronic pollution exposure [156]. Separately, topical short-term exposure assessments showed that treated skin did not exhibit significant increases in heavy metal accumulation compared with placebo-treated areas after 6 h of exposure to a polluted environment, supporting a localized barrier-protective effect during acute pollution challenge [156]. These clinical benefits were significantly greater than those observed with placebo and are mechanistically consistent with the antioxidant and anti-inflammatory actions of Pycnogenol®, which support ECM homeostasis, including hyaluronic acid and collagen synthesis [157]. Across available human studies, the strength of clinical evidence varies with study design, sample size, duration, and the inclusion of mechanistic endpoints. Randomized, placebo-controlled oral trials conducted in polluted environments provide the most consistent improvements in barrier-related outcomes such as hydration and TEWL, whereas topical studies, despite smaller cohorts and shorter durations, offer valuable mechanistic insight by linking clinical benefits to changes in barrier integrity, oxidative stress, and inflammatory markers [150–154]. Clinical findings indicate that TEWL and hydration improvements correlate with preservation of epidermal barrier proteins (filaggrin, loricrin, involucrin) and Nrf2-mediated antioxidant defense, enhancing viscoelasticity and reduced pigmentation align with ECM synthesis (hyaluronic acid/collagen) and ROS scavenging; while attenuated oxidative stress markers reflect MAPK/NF-κB inhibition and AhR modulation. Notably, most trials remain small-to-moderate in size, relatively short in duration (typically 4–12 weeks), and largely confined to highly polluted metropolitan settings, which enhances real-world relevance but also limits cross-study comparability due to heterogeneity in formulations, endpoints, and exposure assessment, underscoring the need for larger, multi-site, longer-term clinical trials with standardized methodologies to strengthen mechanistic and translational interpretation [150–154,156,157].

## 5. Conclusion and future challenges: insight into phytochemical and botanical development for PM2.5-induced skin injury

Chronic exposure to PM2.5 has been associated with skin barrier dysfunction, collagen degradation, and premature aging by modulating redox balance and inflammatory signaling pathways, resulting in molecular alterations in both epidermal keratinocytes and dermal fibroblasts. In light of the detrimental effects of PM2.5 on cutaneous tissues, botanicals and their active phytochemicals, known for their antioxidant, anti-inflammatory, and skin-rejuvenating properties, represent a promising avenue for developing both preventive and therapeutic strategies against pollution-induced skin damage. Phytochemicals have been shown to modulate key signaling pathways, including AhR, AP-1, NF-κB, MAPK, and Nrf2, all of which are central to regulating cellular defense mechanisms in skin cells. Targeting these pathways may be crucial for mitigating oxidative stress, inflammation, skin barrier dysfunction and ECM degradation, thereby addressing the core pathological processes underlying PM2.5-induced skin damage. Nevertheless, translating these protective effects into clinical practice often proves challenging due to the poor bioavailability of many phytochemicals. To address this limitation, researchers are turning to advanced drug delivery systems, such as nanoformulations, which can enhance stability, solubility, and targeted delivery of these compounds to the skin [158]. Due to ethical considerations, direct application of toxic air pollutants to human skin is not feasible, limiting the number of studies that can directly assess cosmetic efficacy under polluted conditions.

Beyond topical interventions, oral supplementation has gained increasing attention as a strategy to protect the skin from environmental stressors. Combining topical and oral delivery can enhance the pharmacokinetic profile and improve tissue penetration of bioactive compounds. Multidisciplinary approaches, including optimized delivery systems and structural analysis of phytochemicals, can further unlock their therapeutic potential. Such strategies hold promises for maximizing the effectiveness of phytochemicals as anti-pollution agents in skin protection. Future research should prioritize the optimization of nanoparticle formulations and advanced delivery systems to enhance the bioavailability, stability, and targeted delivery of bioactive phytochemicals to dermal target sites [159,160]. Well-designed clinical trials combining both topical and oral interventions under controlled pollution exposure are essential to validate the translational efficacy and real-world therapeutic application, targeting pollution-induced skin damage. In addition, systematic investigation into the potential synergistic, additive or antagonistic effects among multiple phytochemical constituents within complex formulations is essential to maximize therapeutic benefits while minimizing adverse effects [161]. Exploring co-delivery systems that combine phytochemicals with conventional drugs may mitigate therapeutic limitations and enhance efficacy through multi-targeted mechanisms [162]. Collectively, these priorities will help establish evidence-based, mechanism-driven, and clinically scalable botanical strategies for preventing and treating pollution-associated skin injury and premature aging.
